# Innate immune receptors over expression correlate with chronic chagasic cardiomyopathy and digestive damage in patients

**DOI:** 10.1371/journal.pntd.0006589

**Published:** 2018-07-25

**Authors:** Nathalie de Sena Pereira, Tamyres Bernadete Dantas Queiroga, Daniela Ferreira Nunes, Cléber de Mesquita Andrade, Manuela Sales Lima Nascimento, Maria Adelaide Do-Valle-Matta, Antônia Cláudia Jácome da Câmara, Lúcia Maria da Cunha Galvão, Paulo Marcos Matta Guedes, Egler Chiari

**Affiliations:** 1 Department of Parasitology, Federal University of Minas Gerais, Minas Gerais, Belo Horizonte, Brazil; 2 Department of Microbiology and Parasitology, Federal University of Rio Grande do Norte, Rio Grande do Norte, Natal, Brazil; 3 School of Health, Potiguar University, Natal, RN, Brazil; 4 Department of Biomedical Sciences, University of Rio Grande do Norte State, Rio Grande do Norte, Mossoró, Brazil; 5 Edmond and Lily Safra International Institute of Neurosciences, Rio Grande do Norte, Macaíba, Brazil; 6 Laboratory of Cellular Ultrastructure, Oswaldo Cruz Institute/FIOCRUZ, Rio de Janeiro, Rio de Janeiro, Brazil; 7 Department of Clinical and Toxicological Analyses, Federal University of Rio Grande do Norte, Natal, Brazil; Albert Einstein College of Medicine, UNITED STATES

## Abstract

Chronic chagasic cardiomyopathy (CCC) is observed in 30% to 50% of the individuals infected by *Trypanosoma cruzi* and heart failure is the important cause of death among patients in the chronic phase of Chagas disease. Although some studies have elucidated the role of adaptive immune responses involving T and B lymphocytes in cardiac pathogenesis, the role of innate immunity receptors such as Toll-like receptors (TLRs) and Nod-like receptors (NLRs) in CCC pathophysiology has not yet been determined. In this study, we evaluated the association among innate immune receptors (TLR1-9 and nucleotide-binding domain-like receptor protein 3/NLRP3), its adapter molecules (Myd88, TRIF, ASC and caspase-1) and cytokines (IL-1β, IL-6, IL-12, IL-18, IL-23, TNF-α, and IFN-β) with clinical manifestation, digestive and cardiac function in patients with different clinical forms of chronic Chagas disease. The TLR8 mRNA expression levels were enhanced in the peripheral blood mononuclear cells (PBMC) from digestive and cardiodigestive patients compared to indeterminate and cardiac patients. Furthermore, mRNA expression of IFN-β (cytokine produced after TLR8 activation) was higher in digestive and cardiodigestive patients when compared to indeterminate. Moreover, there was a positive correlation between TLR8 and IFN-β mRNA expression with sigmoid and rectum size. Cardiac and cardiodigestive patients presented higher TLR2, IL-12 and TNF-α mRNA expression than indeterminate and digestive patients. Moreover, cardiac patients also expressed higher levels of NLRP3, ASC and IL-1β mRNAs than indeterminate patients. In addition, we showed a negative correlation among TLR2, IL-1β, IL-12 and TNF-α levels with left ventricular ejection fraction, and positive correlation between NLRP3 with cardiothoracic index, and TLR2, IL-1β and IL-12 with left ventricular mass index. Together, our data suggest that high expression of innate immune receptors in cardiac and digestive patients may induce an enhancement of cytokine expression and participate of cardiac and digestive dysfunction.

## Author summary

Chronic chagasic cardiomyopathy (CCC) is the main cause of death during *Trypanosoma cruzi* (*T*. *cruzi*) infection in patients. Individuals with CCC have high production of inflammatory mediators such as IFN-γ, TNF-α, IL-1β and nitric oxide (NO) which are involved with myocarditis, fibrosis, and myocardial hypertrophy. Yet the role of innate immunity receptors in CCC pathophysiology has not been addressed. Activation of TLRs and NLRs is fundamental to activate the innate immune system and also to modulate adaptive responses. Herein we have evaluated the association between innate immune receptors and innate cytokines with the clinical manifestation, and with the cardiac function in patients with different clinical forms of chronic Chagas disease. Our data suggest that high TLR2 and NLRP3 expression in cardiac patients may induce an enhancement of proinflammatory cytokine expression such as IL-1β, IL-12, TNF-α and participate of the pathophysiology of CCC. A better understanding of immunological mechanisms involved in CCC may lead to reduced morbidity and mortality associated with the most lethal clinical manifestation of Chagas Disease.

## Introduction

Chagas disease is caused by the *Trypanosoma cruzi* (*T*. *cruzi*) parasite, and affects about 6 million to 7 million people in Latin America; moreover, 1.2 million people have chronic chagasic cardiomyopathy (CCC), which is the main cause of 12,000 deaths annually [1,2]. In the chronic phase of Chagas disease approximately 30 to 50% of patients develop cardiac disease, 5 to 15% develop digestive disease and 2 to 10% develop cardiodigestive form [2–4]. Neuronal depopulation and changes in cardiac and myenteric plexus conduction systems are fundamental for the pathophysiology of CCC, megaesophagus and megacolon. Cardiac conduction system may be diffusely affected from the sinus node to the distal third of His bundle. Chagasic patients with CCC have right bundle branch block (present in 13 to 35% of patients) as an electrocardiographic alteration most frequently suggestive of Chagas' disease, often associated with anterosuperior left bundle branch block [5]. Ventricular extrasystole occurs (15% to 55% of patients) usually isolated, but when complex or associated with other electrocardiographic alterations are correlate with left ventricular systolic and diastolic function, and diastolic diameter [6–8]. The severity of ventricular arrhythmia is often correlated with the degree of left ventricular dysfunction, although some patients with CCC and ventricular tachycardia or ventricular atrial block have preserved global ventricular function [4,6]. Sudden death is more frequent in males (mainly between 30 and 50 years of age). Other electrocardiographic changes observed in Chagas' disease are represented by low voltage of the QRS complex, notches and abnormal thickenings, low amplitude or absence of R wave in precordial derivations [9,10]. Inflammatory process involves fibrosing and progressive chronic myocarditis is also the key substrate for impairment of the conduction system in Chagas disease [5]. Inflammatory cytokines (IL-12, IFN-γ and TNF-α), nitric oxide, autoantibodies, CD8^+^ T lymphocyte are possible correlated with neuronal depopulation [11–15].

Cardiac disease has been correlated with immunological unbalance. Patients with CCC have high production of inflammatory cytokines such as IFN-γ, TNF-α, IL-1β and nitric oxide (NO) which are involved with myocarditis, fibrosis and myocardial hypertrophy. In contrast, asymptomatic patients produce high levels of IL-10 which support control of the inflammatory mechanism in the heart [16–19]. The cardiac form of Chagas disease is related to an increase of T helper (Th) type 1 cells and a decrease of Th2, Th9, Th17, Th22 and regulatory T cell response [11,16]. In fact, exacerbated inflammatory process in cardiac patients has been associated to enhancing the risk of stroke and death [11]. Autoantibodies and CD8^+^ T lymphocytes have also been related to CCC immunopathogenic mechanism [20–22].

TLRs and NLRs are families of pattern recognition receptors (PRRs) located in the plasma membrane, endosomes and cytosol, and are mainly expressed by professional antigen presenting cells (APC), endothelial cells and fibroblasts. PRRs are responsible for recognizing different chemical structures highly conserved in microorganisms known as Pathogen-Associated Molecular Patterns (PAMPs). Signaling through TLRs and NLRs induce the transcription of genes involved in inflammatory response, and its role in the experimental *T*. *cruzi* infection has been investigated. *T*. *cruzi* contains a variety of ligands such as glycosylphosphatidylinositol (GPI) anchors of mucin-like glycoproteins, glycoinositolphospholipid (GIPL) and nucleic acids which activate different PRRs [23–26]. In fact, a deficiency of Myd88, TLR4, TLR7 and TLR9 lead mice to being more susceptible to *T*. *cruzi* infection [27–29]. However, TLR2 signaling and NF-κβ activation induce pro-IL-1β production, which triggers cardiomyocyte hypertrophy in *T*. *cruzi* infected rats [30]. Chagasic patients with a decrease in signal transduction upon ligation of TLR2 or TLR4 to their respective ligand may exhibit low NF-κβ activation and have a low risk of developing CCC [31]. NLRs were extensively characterized as PRRs for bacterial and viral infection [32–34], and their role in recognizing intracellular parasites has been studied. Knockout mice for NOD1 are more susceptible to *T*. *cruzi* infection. Bone marrow-derived macrophages from NOD1 knockout mice show a reduction of products dependent on NF-kB activation and fail to control the infection in the presence of IFN-γ [35]. NLRP3 inflammasome signaling activates apoptosis-associated speck–like protein containing a caspase recruitment domain (ASC) and caspase-1, thereby inducing the cleavage of pro-IL-1β and pro-IL-18 in their active forms [36–38]. ASC inflammasomes are critical determinants of host resistance to infection with *T*. *cruzi*. NLRP3^-/-^, ASC^-/-^ and caspase-1^-/-^ mice exhibit a higher mortality, cardiac parasitism, and myocarditis than wide type mice [39,40]. However, *T*. *cruzi* NLRs agonists are not known.

Although several studies have elucidated the role of TLRs and NLRs in experimental infection by *T*. *cruzi*, the role in human CCC pathophysiology has not yet been determined. The activation of TLRs and NLRs is important in directing adaptive responses, thus resulting in macrophage activation which are important cells involved in heart disease [31,41]. In this study, we have described an increase in several innate components such as NLRP3, ASC, TLR2, IL-1β, IL-12 and TNF-α associated with the pathophysiology of CCC in humans. Moreover, the digestive form of chronic Chagas disease was correlated to high TLR8 and IFN-β mRNA expression. A better understanding of immunological mechanisms involved in CCC may lead to reduced morbidity and mortality associated with the cardiac form of the disease.

## Methods

### Study population and ethics statement

The population was composed of 65 individuals aged between 18 and 79 years old from an endemic area of Chagas disease in Rio Grande do Norte, Northeast, Brazil, as described previously [11]. The individuals were selected using two different serological methods (Chagatest, recombinant ELISA and HAI, and indirect immunofluorescence assay) in accordance with recommendations of the World Health Organization and the Brazilian Consensus of Chagas Disease II [42]. Western blot (TESAcruzi®, BioMérieux, Brazil) confirmatory sorological test was performed [43]. Informed consent was obtained from the participants and the study was approved by the Research Ethics Committee of the State University of Rio Grande do Norte (UERN) under protocol number 027.201, and a Certificate of National System of Ethics in Research (CAEE—SISNEP) with protocol number 0021.0.428.000–11. The study was performed according to human experimental guidelines of the Brazilian Ministry of Health and the Helsinki Declaration.

### Clinical evaluations

Individuals with confirmed positive serology to Chagas disease were clinically evaluated including electrocardiogram (ECG) mapping and chest X-ray, 2D-echocardiogram (ECHO) and 24h Holter examination. Chagasic patients (Table 1) were classified as indeterminate (n = 18), cardiac (n = 17), digestive (n = 15) and cardiodigestive (n = 15) clinical forms, according to the World Health Organization and Brazilian Consensus of Chagas Disease [42]. Uninfected healthy individuals (n = 15) were used as controls. Clinical evaluations were performed in all chagasic patients as previously described [3]. First, plain posteroanterior and lateral chest radiography were performed to evaluate the cardiothoracic index, and which was considered abnormal if attaining a value >0.5 [3]. Esophageal contrast radiography was performed in right anterior oblique position using barium sulfate (Bariogel®, Cristália Laboratory, Brazil) classifying the esophagus changes into four groups [44]. Contrasted colon radiographs were performed in the supine, ventral and right lateral position [45] using barium sulfate solution (Bariogel ®, Cristália Laboratory, Brazil) via the rectum without prior bowel preparation or double contrast use. The sigmoid was classified into four grades (zero to three) according to Silva et al. [46] modified by Andrade and coworkers [3]. Radiographic examinations were performed using radiology equipment with X-ray penetration to deep parts (VMI®, Brazil). Electrocardiographic alterations were determined using a portable EP3 2008 electrocardiograph (Dixtal, Brazil) with three channels and 12-lead; electrocardiographic recording was based on the Minnesota Code modified, adapted for Chagas disease [10]. Next, conventional, parasternal, supra sternal, apical, subcostal transthoracic echocardiogram and its variations were performed in all patients to calculate cardiac dimension and volumes in accordance with the recommendations of the American Society of Echocardiography [47] and using echocardiography with color flow mapping performed in standard views (General Electric Healthcare, USA). The left ventricular ejection fraction (LVEF) was calculated according to the modified Simpson's rule (biplane method) [47]. The left ventricular mass index (LVMI) was calculate by the formula LVMI = heart mass (g)/ patient's body surface (m^2^). Patients with cardiomegaly, electrocardiographic or echocardiographic alterations suggestive of Chagas disease underwent electrocardiographic monitoring for 24 hours (24-Holter) using a Cardiolight Digital Recorder (Cardios, São Paulo, Brazil).

**Table 1:** Clinical data of chronic chagasic patients from the Northeast of Brazil included in this investigation.

		Indeterminate	Cardiac	Cardiodigestive	Digestive
**Number of Patients**		18/65	17/65	15/65	15/65
**Male**		10/18	6/17	10/15	5/15
**Female**		8/18	11/17	5/15	10/15
**Age–years**		41.4 ± 10.7	49.7 ± 11.8	65.0 ± 10.6	57.6 ± 8.9
**Hypertension**		2/18	3/17	7/15	4/15
**Number of patients with Megacolon**		-	-	8/15	9/15
**Number of patients with Megaesophagus**		-	-	3/15	3/15
**Number of patients with Megaesophagus and Megacolon**		-	-	4/15	3/15
**Sigmoid size–centimeter (cm)**		4.41 ± 0.57	4.5 ± 0.71	7.96 ± 2.87	7.72 ± 4.6
**Rectum size—cm**		4.89 ± 0.81	5.72 ± 0.89	6.36 ± 1.89	7.5 ± 3.1
**Left ventricular ejection fraction ±standard deviation**		64.6 ± 3.42	55.8 ± 14.96	56.2 ± 13.84	65.0 ± 6.48
**Cardiothoracic index± standard deviation**		0.43 ± 0.05	0.48 ± 0.05	0.50 ± 0.05	0.42± 0.03
**Left ventricular mass index ± standard deviation**		97.6 ± 21.6	100.6 ± 19.8	126.5 ± 54.4	95.9 ±16.8
**Left ventricular diastolic diameter ± standard deviation**		49.4 ± 2.9	50.6 ± 6.8	51.0 ± 5.8	47.6 ± 3.6
**Right Branch Block (number of patients/%)**		0 (0%)	5 (29.5%)	4 (26.8%)	0 (0%)
**Left Branch Block**		0 (0%)	1 (5.9%)	1 (6.7%)	0 (0%)
**Anterosuperior divisional block**		0 (0%)	4 (23.6%)	3(20.1%)	0 (0%)
**Atrioventricular Block**		1 (5.6%)	3 (17.7%)	2 (13.4%)	1 (6.7%)
**Supraventricular extrasystoles**		1 (5.6%)	1 (5.9%)	1 (6.7%)	0 (0%)
**Ventricular extrasystoles**		0 (0%)	3 (17.7%)	4 (26.8%)	0 (0%)
**Ventricular repolarization change**		1 (5.6%)	3 (17.7%)	1 (6.7%)	1 (6.7%)
**Low voltage of the QRS**		0 (0%)	8 (47.2%)	1 (6.7%)	2 (13.4%)

### Real time PCR

Innate immune receptors (TLR1, TLR2, TLR3, TLR4, TLR5, TLR6, TLR7, TLR8, TLR9 and NLRP3), signaling molecules (Myd88, TRIF, ASC, Caspase-1) and cytokine (IL-1β, IL-6, IL- 12, IL-18 and TNF-α) mRNA expression were detected by Real-Time PCR (qPCR) in peripheral blood mononuclear cells (PBMC) obtained from chagasic patients. Total RNA was obtained using Trizol reagent (Invitrogen™, Carlsbad, CA, USA) and SV Total RNA Isolation System (Promega, Madison, WI, USA) with DNase treatment step. cDNA synthesis was performed with the High Capacity cDNA Reverse Transcription kit (Applied Biosystems, USA) using the Eppendorf Mastercycler gradient set (Eppendorf, USA). The qPCR reactions were performed using SYBR Green (Applied Biosystems, USA) supported by 7500 Fast Real time thermocycler (Applied Biosystems, Warrington, USA). The reactions were performed in 96 well plates (MicroAmp®, Applied Biosystems, USA) and the standard PCR conditions were as follows: 50°C (2 min) and 95°C (10 min) followed by 40 cycles of 94°C (30 s), variable annealing primer temperature (Table 2) (30 s), and 72°C (1 min). Specific primers (Table 2) were obtained by the Primer Express software (Applied Biosystems, USA). The mRNA expression levels of the innate immune receptors, adapter molecules and cytokines were determined using the mean Ct values from triplicate measurements to calculate the relative expression levels of the target genes in the Chagas disease patients compared to healthy controls, and were normalized to the housekeeping gene β-actin using the 2^–ΔΔCt^ formula.

**Table 2:** Sequences of the primers used for RT-PCR reactions.

Target	Sense and Antisense sequences	Primer annealing temperature
β-actin	TGACTCAGGATTTAAAAACTGGAA GCCACATTGTGAACTTTGGG	56.5°C
TLR1	GGTACCAGGCCCTCTTCCTCGTTAG TAGGAACGTGGATGAGACCGTTTTT	63.6°C
TLR2	GTTGCAAGCAGGATCCAAAGGAGAC GCAGATACCATTGCGGTCACAAGAC	63.6°C
TLR3	TGGGTCTGGGAACATTTCTCTTC TGAGATTTAAACATTCCTCTTCGC	58.5°C
TLR4	TGAATTTCTACAAAATCCCCGACAA AGAGGTGGCTTAGGCTCTGATATGC	60.3°C
TLR5	GCTGGACTGCAGTGACACAATCTC GAGAAGCCACGTTGTCAGTAGCATC	63.3°C
TLR6	GCAAAAACCCTTCACCTTGTTTTTC CCAAGTCGTTTCTATGTGGTTGAGG	60.3°C
TLR7	TTTACCTGGATGGAAACCAGCTA TCAAGGCTGAGAAGCTGTAAGCTA	59.3°C
TLR8	TTATGTGTTCCAGGAACTCAGAGAA TAATACCCAAGTTGATGATCGATAAGTTTG	59.0°C
TLR9	CCACCCTGGAAGAGCTAAACC GCCGTCCATGAATAGGAAGC	60.8°C
Myd88	CAAGTACAAGGCAATGAAGAAAG AAGGCGAGTCCAGAACCA	56.9°C
TRIF	ACTGAACGCAGCCTACTCAGC ATGACATGTGGCTCCCAAAAG	59.9°C
NLRP3	GCGATCAACAGGAGAGACCTTTA GCTGTCTTCCTGGCATATCACA	60.1°C
ASC	CTGGAGCCATGGGGCGCGCG CGGAGTGTTGCTGGGAAGGAG	66.9°C
Caspase-1	GAAGGCATTTGTGGGAAGAA CATCTGGCTGCTCAAATGAA	55.7°C
TNF-α	TTCTGGCTCAAAAAGAGAATTG TGGTGGTCTTGTTGCTTAAAG	55.2°C
IL-1β	GCACGATGCACCTGTACGAT AGACATCACCAAGCTTTTTTGCT	58.2°C
IL-6	CAAATTCGGTACATCCTCGA TGCTGCTTTCACACATGTTACT	56.0°C
IL-12	CACTCCCAAAACCTGCTGAG TCTCTTCAGAAGTGCAAGGGTA	59.0°C
IL-18	AGGAATAAAGATGGCTGCTGAAC GCTCACCACAACCTCTACCTCC	61.0°C
IL-23	TTCTGCTTGCAAAGGATCCA AATATCCGATCCTAGCAGCTTCTC	58.0°C
IFN-α	GAAGAATCTCTCCTTTCTCCTGCC ATGGAGGACAGAGATGGCTTG	61.0°C
IFN-β	TGCCCTAAGGACAGGATGAAC GCGTCCTCCTTCTGGAACTG	60.9°C

### Enzyme linked immunosorbent assay (ELISA)

Cytokine quantification was performed in sera from indeterminate (n = 18), cardiac (n = 17), cardiodigestive (n = 15) and digestive (n = 15) chagasic patients. Uninfected individuals were used as a control (n = 15). The ELISA sets were IL-1β, IL-12 (p70) and TNF-α (BD OptEIA^TM^, BD Bioscience), and procedures were performed according to the manufactures`instructions. Optical densities were measure at 450ηm.

### Statistical analysis

Data are reported as mean ± standard deviation (SD). Kolmogorov-Smirnov test was used to verify parametric or non-parametric data distribution. The mRNA expression levels were compared using the Kruskal-Wallis test. Correlations among left ventricular ejection fraction, esophagus and colon dilation, innate immune receptors and cytokines were performed using the Spearman test. Differences were considered significant when p <0.05. Our analyses were performed using PRISM 5.0 software (GraphPad, CA, USA).

## Results

### Clinical evaluations

Chagasic patients (n = 65) were classified as indeterminate (n = 18), cardiac (n = 17), digestive (n = 15) and cardiodigestive (n = 15) clinical forms. Chest X-ray demonstrated cardiomegaly in approximately 10% of cardiac and cardiodigestive patients. Electrocardiographic changes were not always associated with cardiac symptoms. Three cardiac patients had right bundle branch block, two also had anterosuperior divisional block. Four cardiodigestive patients presented right bundle branch block, two also presented anterosuperior divisional block. All ventricular atrial blocks were first degree, except for one patient with ventricular atrial blockade who received pacemaker implantation. The echocardiogram showed similar diastolic diameters and left ventricular mass index in indeterminate, cardiac, digestive and cardiodigestive chagasic patients (Table 1).

### Chronic chagasic cardiomyopathy is correlated with high TLR2, IL-12 and TNF-α expression

**Fig 1:**
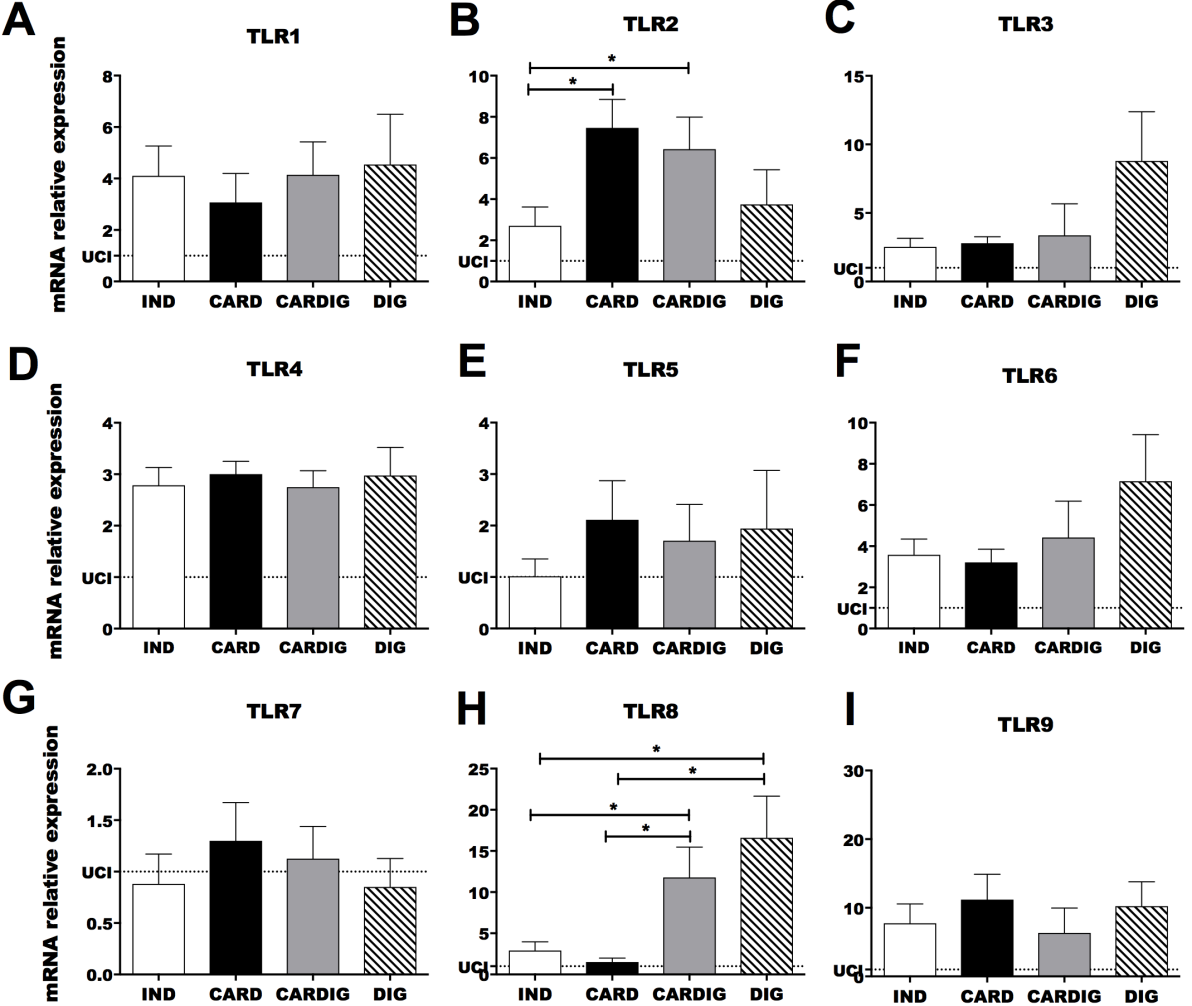
Chronic chagasic cardiomyopathy is correlated with high TLR2 mRNA expression and digestive form is correlated with high TLR8 expression. The mRNA expression levels of TLR1 (A), TLR2 (B), TLR3 (C), TLR4 (D), TLR5 (E), TLR6 (F), TLR7 (G), TLR8 (H) and TLR9 (I) were determined by real-time PCR in peripheral blood mononuclear cells of patients with the indeterminate (n = 18), cardiac (n = 17), cardiodigestive (n = 15) and digestive (n = 15) clinical forms of Chagas disease. The expression levels were normalized to the expression level of β-actin. The results are expressed as the means ± standard errors. *p < 0.05. UCI: Uninfected control individuals (n = 15).

**Fig 2:**
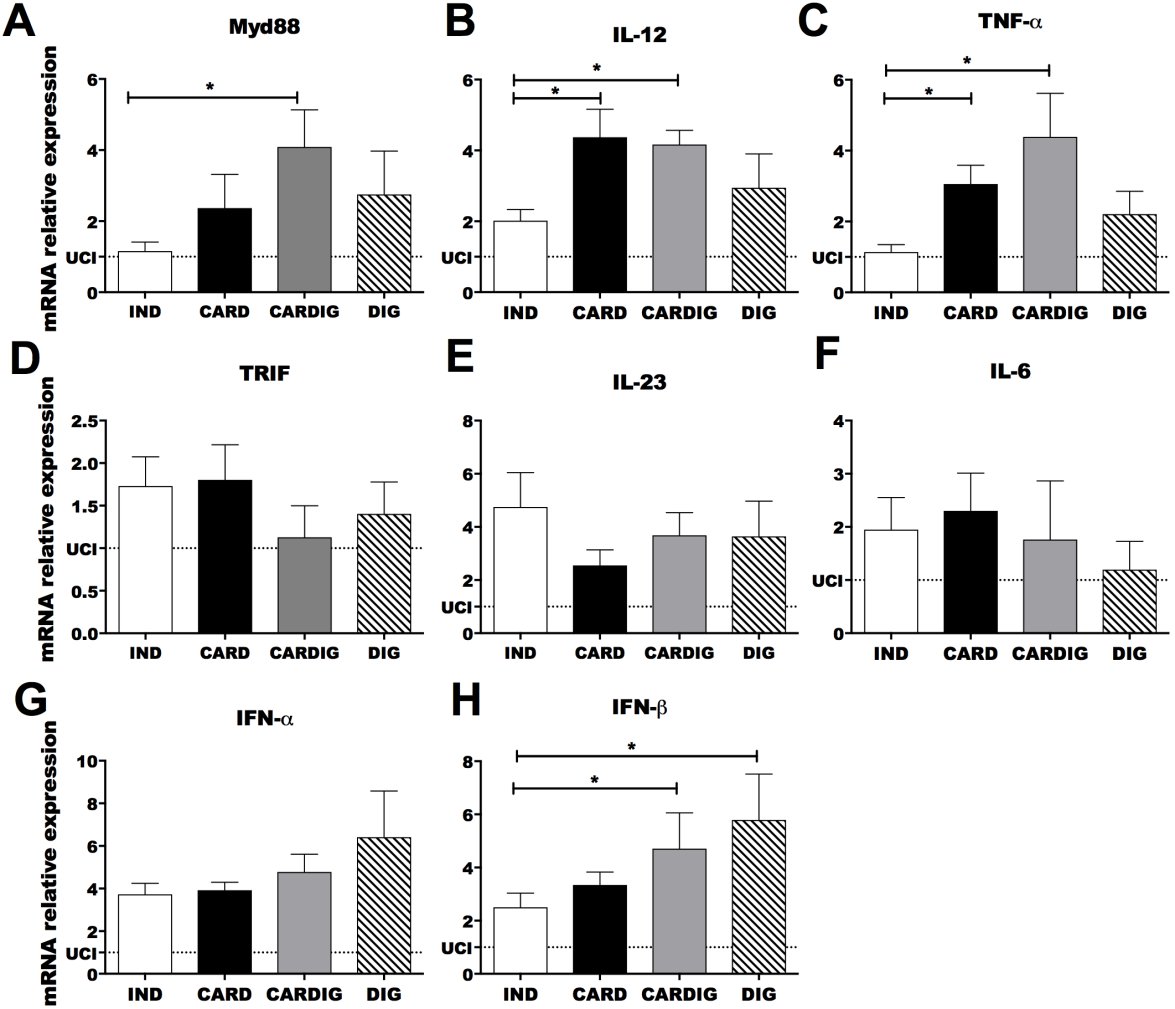
Chronic chagasic cardiomyopathy is correlated with high IL-12, TNF-α and IFN-β mRNA expression. The mRNA expression levels of Myd88 (A), IL-12 (B), TNF-α (C), TRIF (D), IL-23 (E), IL-6 (F), IFN-α (G) and IFN-β (H) were determined by real-time PCR in peripheral blood mononuclear cells of patients with the indeterminate (n = 18), cardiac (n = 17), digestive (n = 15) and cardiodigestive (n = 15) clinical forms of Chagas disease. The expression levels were normalized to the expression level of β-actin. The results are expressed as the means ± standard errors. *p < 0.05. UCI: Uninfected control individuals (n = 15).

**Fig 3:**
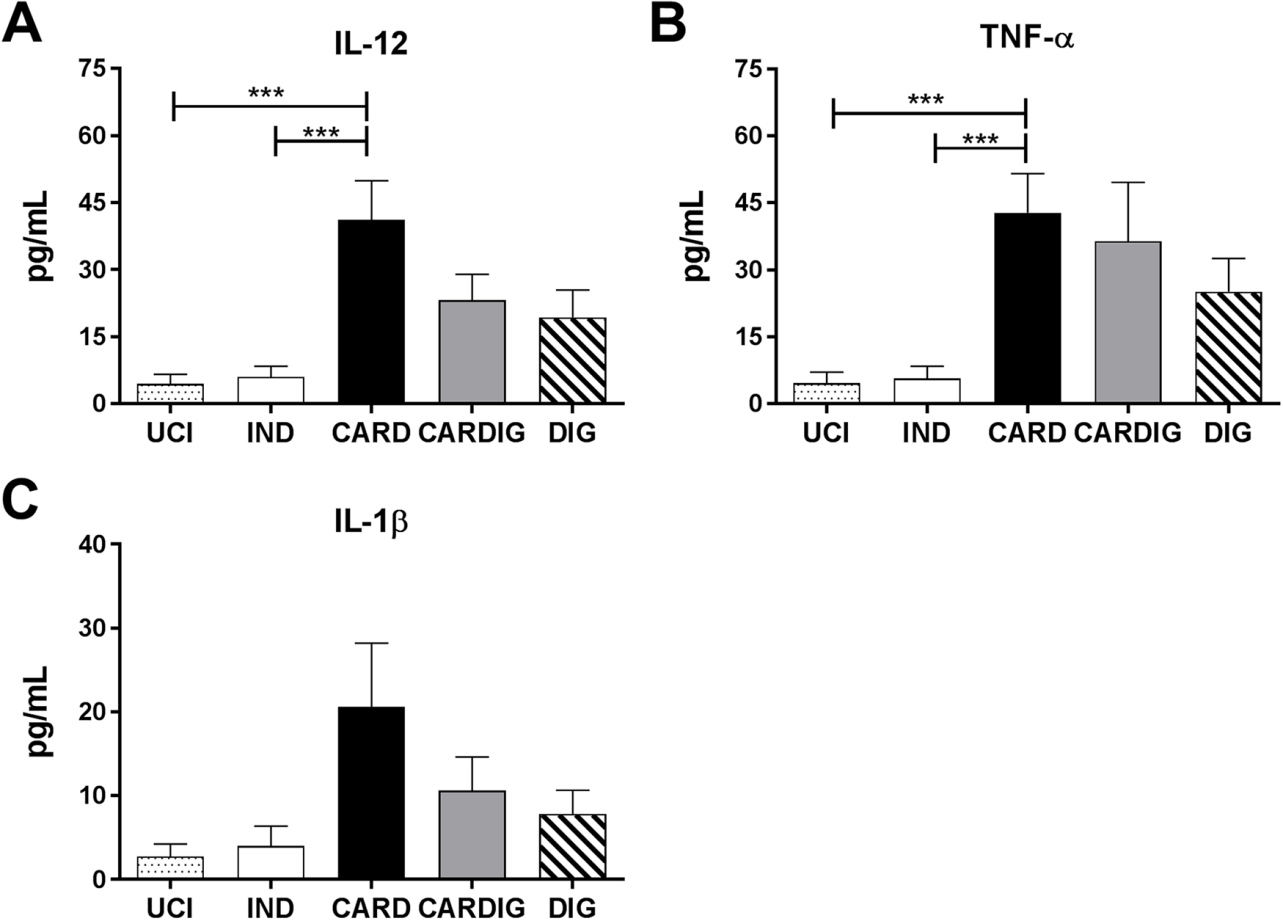
Chronic chagasic cardiomyopathy is correlated with high IL-12 and TNF-α levels in sera. Levels of the cytokines IL-12 (A), TNF-α (B) and IL-1β (C) was analyzed by ELISA in the sera of indeterminate/IND (n = 18), cardiac/CARD (n = 17), digestive/DIG (n = 15) and cardiodigestive/CARDIG (n = 15) patients. The results are expressed as means ± standard errors. *p < 0.05. UCI: Uninfected control individuals (n = 15).

In an attempt to elucidate the inflammatory mechanism involved in CCC development we analyzed the mRNA expression of innate immune receptors in chagasic individuals grouped according to clinical forms as indeterminate, cardiac, digestive and cardiodigestive (Table 1). Patients with different clinical manifestations of Chagas disease showed similar expression of TLR1, TLR3, TLR4, TLR5, TLR6, TLR7 and TLR9 mRNA (Fig 1). Interestingly, cardiac and cardiodigestive patients presented higher TLR2 mRNA expression than indeterminate and digestive patients (Fig 1B). Furthermore, cardiodigestive patients presented higher Myd88 mRNA expression than indeterminate and cardiac patients (Fig 2A). Cardiac patients showed higher mRNA expression of IL-12 and TNF-α transcripts (cytokines produced upon TLR activation) than indeterminate patients (Fig 2B and 2C). We observed similar expression of TRIF, IL-6, IL-23 and IFN-α in chagasic patients with different clinical manifestations of Chagas disease (Fig 2D–2G). Moreover, there was higher production of inflammatory cytokines (TNF-α and IL-12) induced by the TLRs activation in sera in cardiac patients than in indeterminate and uninfected controls (Fig 3A and 3B). However, no significant difference was observed between the levels of IL-1β between the different groups of patients (Fig 3C). Together, these data indicate that TLR2 expression in cardiac patients may induce an enhancement of IL-12 and TNF-α expression and correlate to cardiac dysfunction.

### Digestive and cardiodigestive clinical forms are correlated with high TLR8 and IFN-β mRNA expression

**Fig 4:**
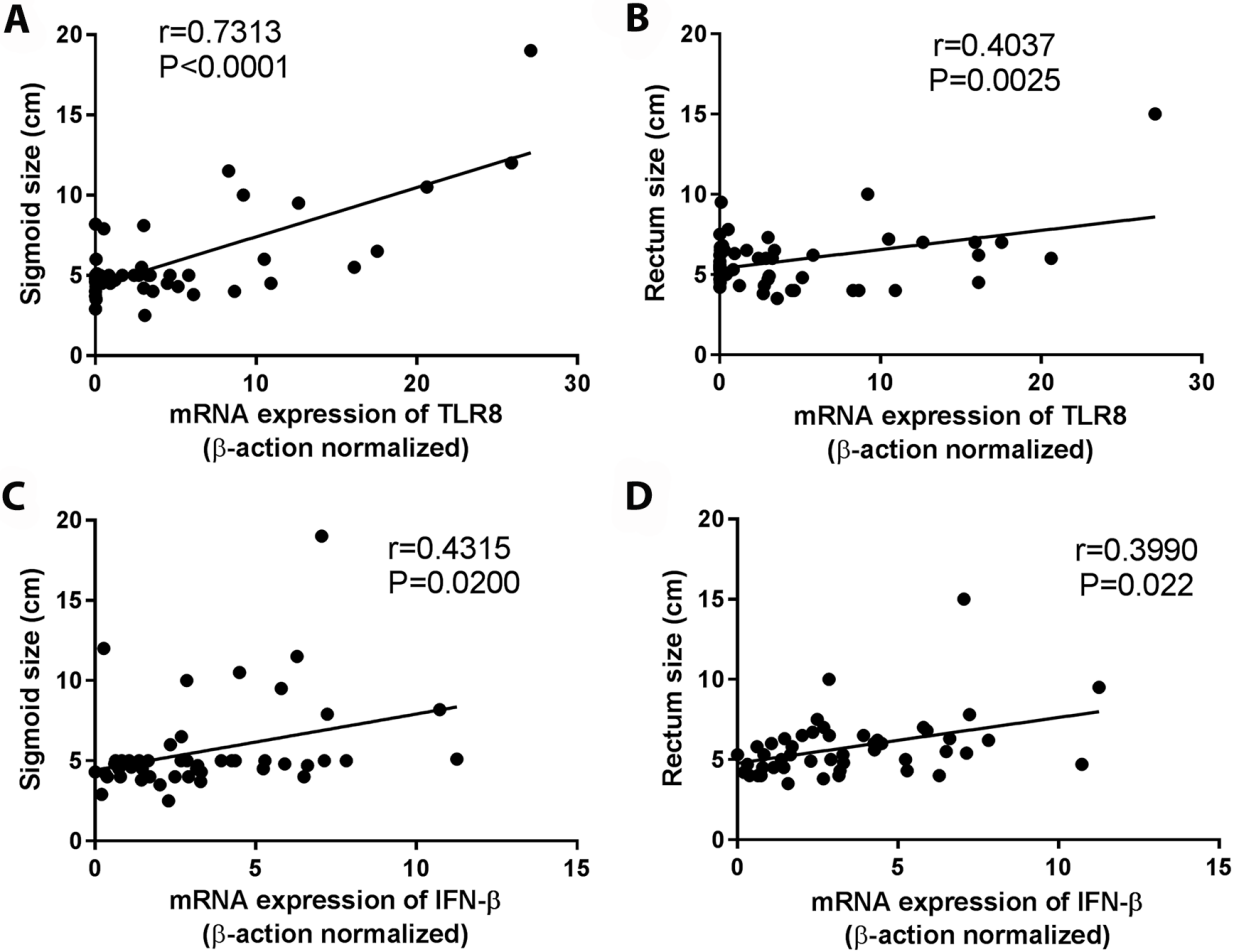
High TLR8 and IFN-β expression are correlated with digestive form of Chagas disease. The mRNA expression levels of TLR8 (A and B) and IFN-β (C and D) were determined by real-time PCR in peripheral blood mononuclear cells of patients with the indeterminate (n = 18), cardiac (n = 17), digestive (n = 15) and cardiodigestive (n = 15) clinical forms of Chagas disease and correlated with sigmoid and rectum size. The expression levels were normalized to the expression level of β-actin. Spearman test was used.

The mRNA expression of TLR8 was enhanced in digestive and cardiodigestive patients compared to indeterminate and cardiac patients. Furthermore, the mRNA expression of IFN-β (cytokine produced after TLR8 activation) was higher in digestive and cardiodigestive patients when compared with indeterminate (Fig 2H). In attempt to evaluate the TLR8 and IFN-β participation in the development of the digestive form of Chagas disease, we analyzed the correlation between the mRNA expression of TLR8 and IFN-β with the rectum and sigmoid size, resulting in a positive correlation observed between TLR80020and IFN-β and rectum and sigmoid size (Fig 4A–4D).

### Enhanced expression of NLRP3, ASC and IL-1β transcripts correlates with the chronic chagasic cardiomyopathy in patients

**Fig 5:**
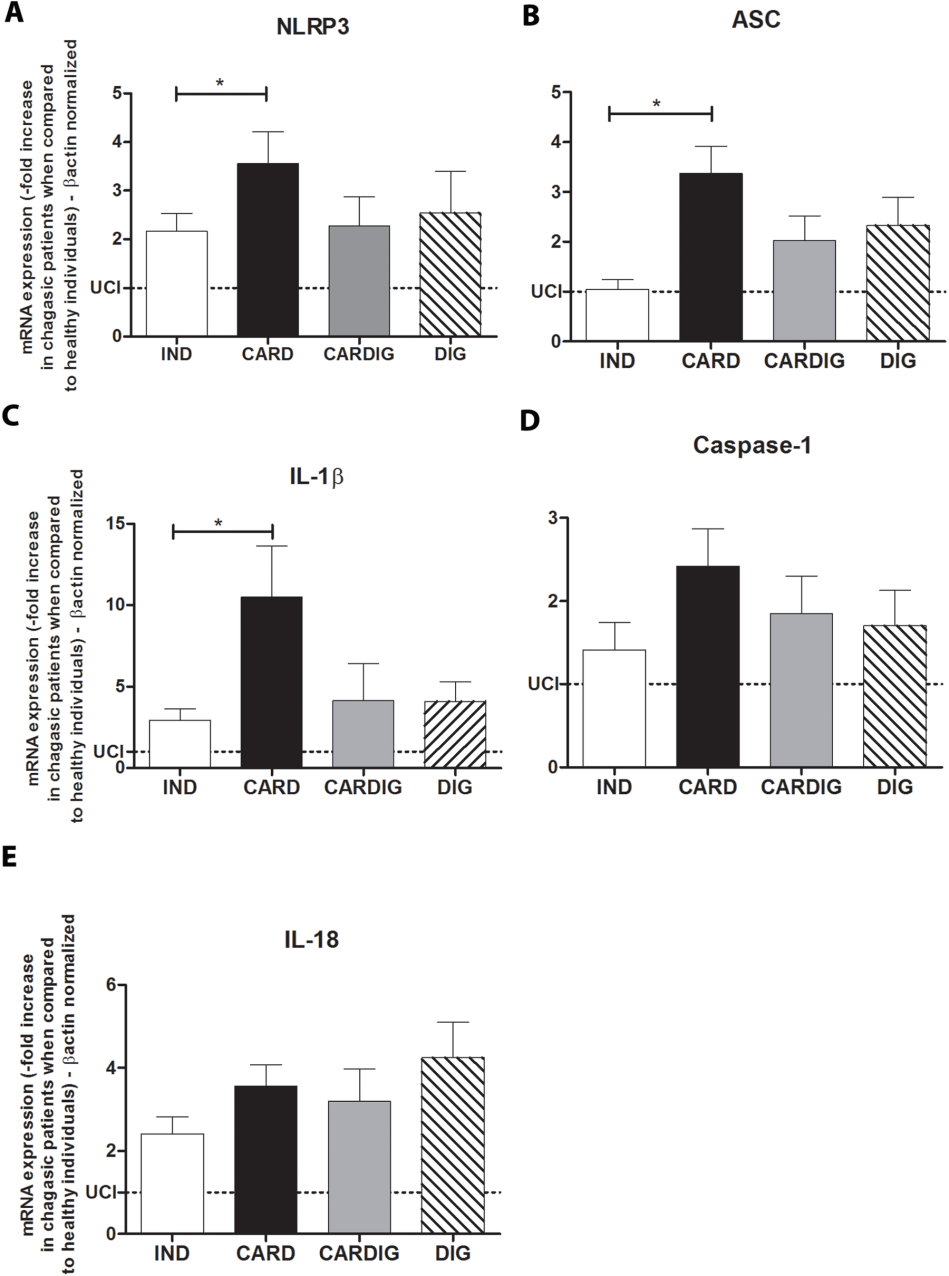
Cardiac patients exhibited higher NLRP3, ASC and IL-1β mRNA expression than indeterminate patients. The mRNA expression levels of NLRP3 (A), ASC (B), Caspase-1 (C), IL-1β (D) and IL-18 (E) were determined by real-time PCR in peripheral blood mononuclear cells of patients with the indeterminate (n = 18), cardiac (n = 17), digestive (n = 15) and cardiodigestive (n = 15) clinical forms of Chagas disease. The expression levels were normalized to the expression level of β-actin. The results are expressed as the means ± standard errors. *p < 0.05. UC: Uninfected control individuals (n = 15).

**Fig 6:**
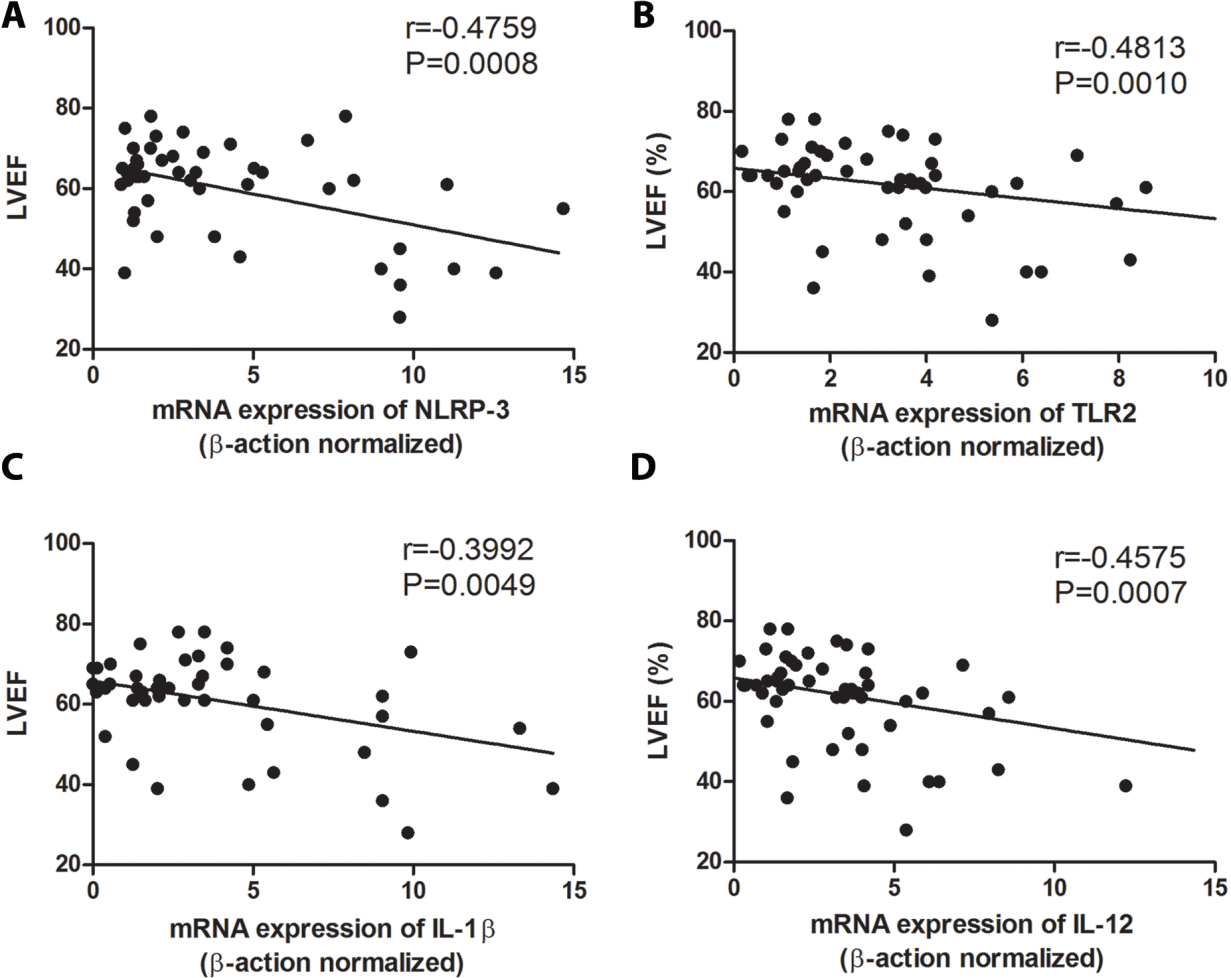
High NLRP3, TLR2, IL-12 and IL-1β expression are correlated with low left ventricular ejection fraction (LVEF). The mRNA expression levels of NLRP3 (A), TLR2 (B), IL-1β (C) and IL-12 (D) were determined by real-time PCR in peripheral blood mononuclear cells of patients with the indeterminate (n = 18), cardiac (n = 17), digestive (n = 15) and cardiodigestive (n = 15) clinical forms of Chagas disease and correlated with left ventricular ejection fraction (LVEF). The expression levels were normalized to the expression level of β-actin. Spearman test was used.

**Fig 7:**
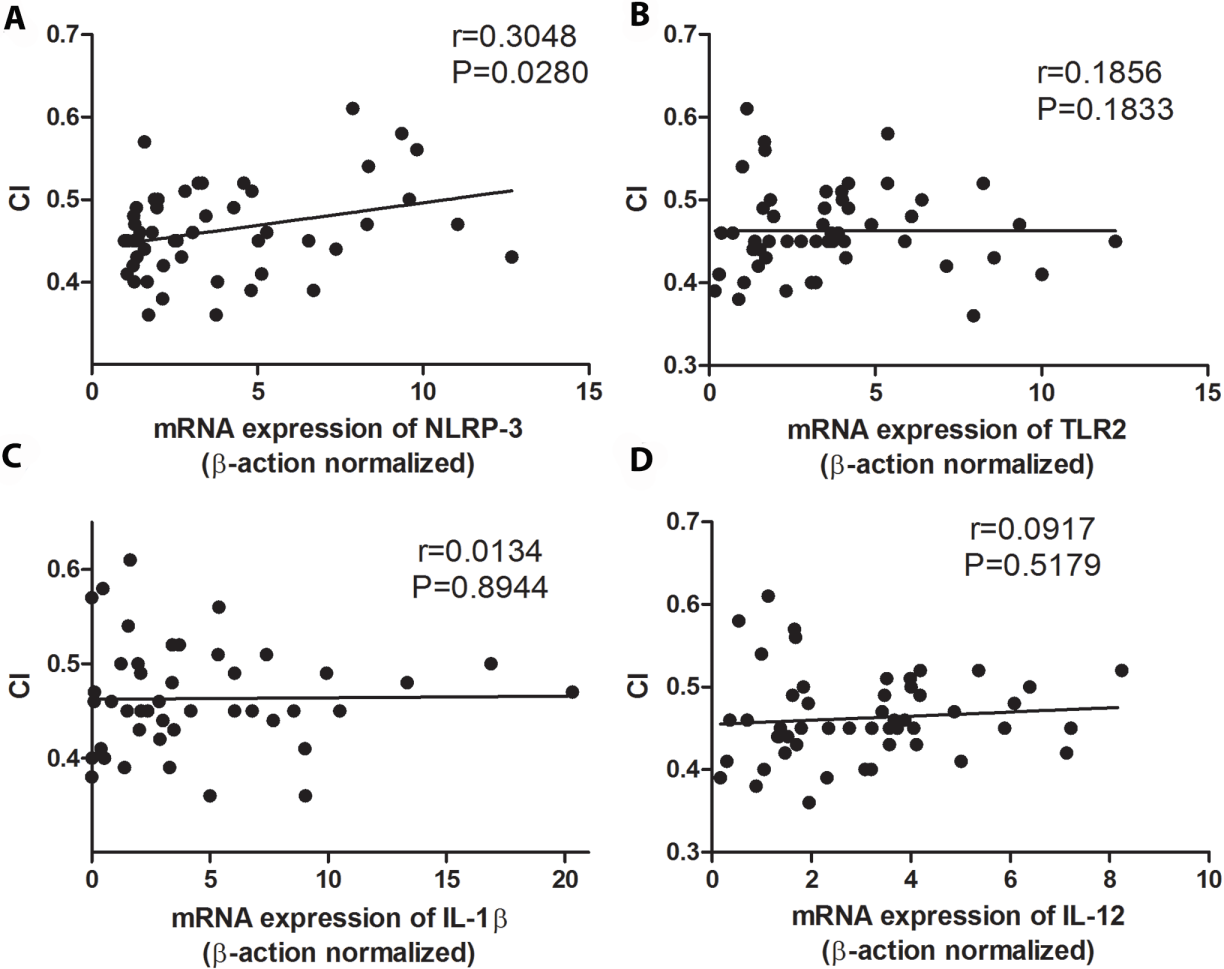
High NLRP3 expression is correlated with high cardiothoracic index (CI). The mRNA expression levels of NLRP3 (A), TLR2 (B), IL-1β (C) and IL-12 (D) were determined by real-time PCR in peripheral blood mononuclear cells of patients with the indeterminate (n = 18), cardiac (n = 17), digestive (n = 15) and cardiodigestive (n = 15) clinical forms of Chagas disease and correlated with cardiothoracic index (CI). The expression levels were normalized to the expression level of β-actin. Spearman test was used.

**Fig 8:**
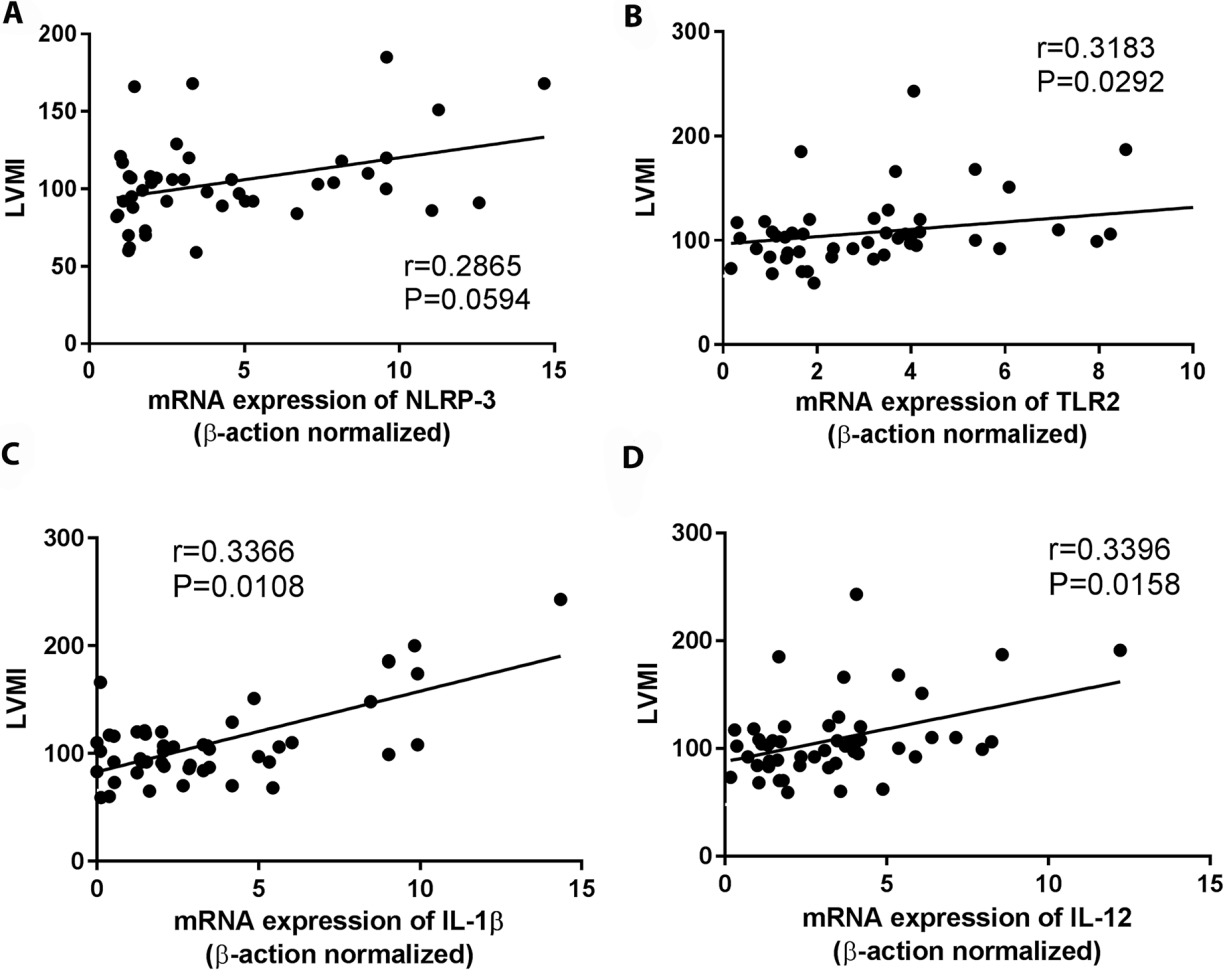
High left ventricle mass index (LVMI) is correlated with high TLR-2, IL-1β and TNF-α mRNA expression. The mRNA expression levels of NLRP3 (A), TLR2 (B), IL-1β (C) and IL-12 (D) were determined by real-time PCR in PBMC of indeterminate (n = 18), cardiac (n = 17), digestive (n = 15) and cardiodigestive (n = 15) patients and correlated with LVMI. Spearman test was used.

We subsequently analyzed the expression of NLRP3 inflammossome, its signaling molecules (ASC and caspase-1) and cytokines produced after its activation (such as IL-1β and IL-18). Cardiac patients showed relevantly higher mRNA expression of NLRP3, ASC and IL-1β than indeterminate patients (Fig 5A–5C). Similar levels of caspase-1 and IL-18 mRNA expression were observed in patients with different clinical forms of chronic Chagas disease (Fig 5D and 5E). We then posteriorly analyzed the correlation between the mRNA expression of TLR2, NLRP3, IL-1β, IL-12 and TNF-α with the left ventricular ejection fraction (LVEF) and cardiothoracic index (CI). We found a negative correlation among NLRP3, TLR2, IL-12 and IL-1β mRNA expression with LVEF (Fig 6A–6D), and positive correlation between NLRP3 with CI (Fig 7A). No correlation was observed between TLR2, IL-1β and IL-12 with CI (Fig 7B–7D), and between NLRP3 with left ventricular mass index (LVMI) (Fig 8A). We also observed a positive correlation between LVMI with TLR2, IL-1β and IL-12 (Fig 8B–8D).

## Discussion

Pathophysiological mechanisms involved in the development of chronic chagasic cardiomyopathy (CCC) have been studied in chagasic patients and several immunopathogenic mechanisms involving the participation of adaptive immune response such as CD4^+^ T helper response [11,16–18,48,49], CD8^+^ T cells [50–53], and autoantibodies production [20,21,54–56] have been elucidated. However, the role of innate immunity receptors in the CCC pathophysiology has not been elicited. In this study, we have assessed the expression of Toll-like Receptors and Nod-like Receptors, their adapter molecules and induced cytokines in cardiac patients, and compared them to indeterminate, digestive and cardiodigestive clinical forms of the disease.

We initially analyzed the mRNA expression of TLRs in PBMCs obtained from the same chagasic patients described in previous study [11]. Patients who showed digestive and cardiodigestive clinical forms presented higher TLR8 mRNA expression when compared to cardiac and indeterminate patients. Human TLR8 recognizes single-stranded RNAs from RNA viruses, as well as detecting RNAs from bacteria in endosomes of dendritic cells [57]. Patients with the digestive clinical form are mainly characterized by the presence of a megaesophagus and megacolon which are caused by the destruction of intramural autonomic ganglia [58]. Gastrointestinal dysfunction can change the feed flow and is associated with bowel inflammatory lesions which distort epithelium gastrointestinal homeostasis, which in turn could allow bacteria penetration and TLR8 activation. This phenomenon could be associated with the development of digestive pathology in chronic chagasic patients. In fact, patients with ulcerative colitis have higher TLR8 mRNA in colon biopsies than healthy subjects, probably due to bacterial RNA of gut microbiota resulting from microbiota dysbiosis [59,60]. Intestinal inflammation intensity in the ulcerative colitis is positively correlated with TLR8 and inflammatory cytokines such as IL-6 and TNF-α [60]. Susceptibility to Crohn's disease (another intestinal inflammatory disease) has also been associated to high TLR8 levels [61,62]. TLR8 activation induces pro-inflammatory cytokine production such as IL-1β, IFN-α, IFN-β, TNF-α, IL-6, and IL-12 in PBMCs, monocytes and dendritic cells in patients [57,63]. Furthermore, in this study we observed higher mRNA expression of TLR8 and IFN-β in digestive and cardiodigestive patients when compared to indeterminate patients. Pathophysiologic alterations in the digestive system during Chagas disease result from the destruction of the enteric nervous system, mainly Auerbach's myenteric plexus. The inflammatory process around the neurons leads to degenerative phenomena, thereby reducing nervous cell numbers and leading to the development of megacolon and megaesophagus [64–66].

Cardiac and cardiodigestive patients showed higher TLR2 mRNA expression than indeterminate and digestive patients. On the other hand, patients with different clinical manifestations of Chagas disease showed similar mRNA expression levels of TLR1, TLR3, TLR4, TLR5, TLR6, TLR7 and TLR9. Literature data has demonstrated that *T*. *cruzi-*infected individuals who have indeterminate clinical form of Chagas disease are heterozygous for the *MAL/TIRAP* S180L variant that leads to a decrease in signal transduction upon ligation of TLR2 or TLR4, probably leading to reduced inflammatory response in the heart [31,67]. Thus, low TLR2 and TLR4 signaling have been associated with a lower risk of developing CCC. TLR2 and TLR4 activations in dendritic cells and macrophages conducing Myd88 and TRIF signaling activate NF-kB and lead to the production of pro-inflammatory cytokines such as IL-6, IL12 and TNF-α [57]. CCC development has been correlated to immunological imbalance involving high IFN-γ and TNF-α production associated with low IL-10 and IL-17 secretion [11,16–18,49,68]. In our study we also observed that cardiac patients showed higher mRNA expression of NLRP3, ASC, CASPASE-1, IL-1β, IL-12 and TNF-α than indeterminate patients. Furthermore, a negative correlation among TLR2, NLRP3, IL-1β and TNF-β mRNA expression with LVEF, and positive correlation of NLRP3 mRNA expression with CI was observed in chagasic patients. NLRP3 inflammasome and apoptosis-associated speck–like protein containing a caspase recruitment domain (ASC) activates caspase-1 in experimental *T*. *cruzi* infection, and induce the production of active IL-1β and IL-18 [39]. Pro-inflammatory cytokines (IL-1β, IL-6 and TNF-β) regulate cell death of inflammatory tissues, modify vascular endothelial permeability, recruit blood cells to inflamed tissues, and induce the production of acute-phase proteins [69]. NLRP3 inflammasome is activated by prokaryotic RNA and different agents that trigger damage-associated molecular patterns (DAMPs) such as UVB irradiation [70] pore-forming toxins [71], urate crystals and silica [72]. Moreover, several host-derived molecules indicative of damage activate the NLRP3 inflammasome, including reactive oxygen species [73], extracellular ATP adenosine [74], uric acid [75,76] and hyaluronan [77] which are released by injured cells. Thus, the NLRP3 activation mechanism observed during experimental *T*. *cruzi* infection [39] by an possible unknown parasite ligand may act together with DAMPs generated by injured cells [69], thereby participating in the immunophysiological mechanisms involved in CCC development in chronic chagasic patients. Pro-inflammatory cytokines such as TNF-α, TNF-β, IL-1α, IL1β, IL-6, IFN-α, IFN-γ and IL-8 induce acute phase protein productions which can opsonize parasites, activate complements, recruit immune cells and induce enzymes which degrade the extra cellular matrix. Acute phase proteins such as C-reactive protein, Serum amyloid A, Serum amyloid P component, Complement factors, Mannan-binding lectin, Fibrinogen, prothrombin, Plasminogen, Alpha 2-macroglobulin, Ferritin, Ceruloplasmin, Haptoglobin, Alpha 1-antitrypsin and α1-antichymotrypsin enhance the inflammatory process [78]. Increased levels of acute phase proteins are associated with increased risk for cardiovascular events in healthy individuals and coronary heart disease patients [79]. C-reactive protein levels are non-specific markers of systemic inflammatory processes, which reflect a vascular inflammation state and they are associated with cardiovascular damage [80]. High C-reactive protein levels have been described in non-chagasic cardiomyopathy [81] and also during CCC [82–85]. Thus, NLRP3 and TLR2 recognize parasite antigens and molecules associated with cell damage, leading to an inflammatory process with cytokines and other inflammatory mediator production that might participate of CCC development.

CCC development is initiated by the presence of the parasite causing cardiomyocyte destruction due to parasite multiplication and inflammation [86]. However, CCC development depends on the parasite’s genetics [87–89] and the genetic background of the patients [67,90–92] which can induce different cardiac tropism patterns by the parasite and influence the immune response [87,93,94]. CCC involves persistent myocarditis, development of conduction disturbances, dysautonomia, cardiomegaly, fibrosis, ventricular wall thinning, microvascular damage, increased platelet activity, microthrombi, myocytolysis, myocardial fibrosis and death [4,95,96]. Persistent myocarditis is responsible for progressive neuronal damage, microcirculatory alterations, heart matrix deformations and consequent organ failure [97]. In this context the important inflammatory process in the myocardium which is responsible for CCC development seems to also be maintained by the innate immunity receptor activation such as TLR2 and NLRP3, which induce the production of inflammatory cytokines (IL-1β, IL-12 and TNF-α), thus amplifying the described inflammatory mechanisms and involving elements of adaptive immunity components such as T CD4 and CD8 lymphocytes and antibodies [57]. Our findings suggest that a high TLR2 and NLRP3 expression in chagasic cardiac patients may induce an enhancement of IL-1β, IL-12, and TNF-α, thereby increasing cardiac inflammation and contributing to the heart dysfunction. One limitation of this study concerns on the widely clinical presentation of patients with CCC, according to the extent of myocardial damage and the relative small sample size. Untreated patients samples are very rare and difficult to obtain but are essential for the understanding of immunological mechanisms of Chagas disease pathophysiology. A better knowledge of the immune response involved in CCC development, the main factor correlated to death related to Chagas disease, may also contribute to reducing mortality and morbidity. The present study generated important data about the disease pathophysiology understanding, suggesting that distinct pattern recognition receptors may contribute differentially to the development of clinical forms of Chagas disease.
